# Supplementary material for: Linear Capacitive Pressure Sensor with Gradient Architecture through Laser Ablation on MWCNT/Ecoflex Film
Source: Polymers (Basel). 2024 Apr 2;16(7):962. doi: 10.3390/polym16070962 (PMC11013779; doi:10.3390/polym16070962)
Supplement: Supplementary file 1 [file polymers-16-00962-s001.zip › polymers-2900735-supplementary.pdf]

Supporting Information

Linear Capacitive Pressure Sensor with Gradient Architecture through Laser Ablation on MWCNT/Ecoflex Film

Chenkai Jiang [1,2] and Bin Sheng [1,2,*]

[1] School of Optical-Electrical and Computer Engineering, University of Shanghai for Science and Technology, Shanghai 200093, China; 15003438539@163.com
[2] Shanghai Key Laboratory of Modern Optical Systems, Engineering Research Center of Optical Instruments and Systems, Shanghai 200093, China
* Correspondence: bsheng@usst.edu.cn

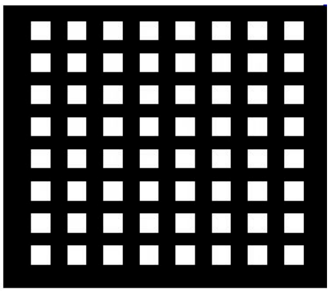

Figure S1. Design drawing of CO_2 laser ablation array (black areas are ablated areas)

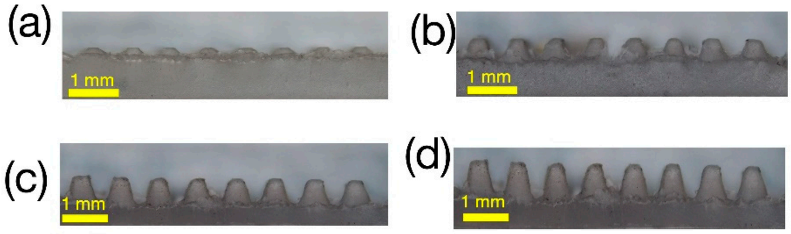

Figure S2. Side view of the Ecoflex micro-cones using the parameters of table S1

Table S1. CO_2 laser ablation parameters.

Number of laser ablations	Laser power(W)	Laser Speed(mm/s)	Microstructure height(μm)
1	20	200	140±20
2	20	200	327±15
3	20	200	500±17
4	20	200	730±21

Table S2 CO_2 laser ablation parameters

Number of laser ablations	Laser power(W)	Laser Speed(mm/s)	Microstructure height(μm)
1	40.0	200	290 ± 21
2	40.0	200	490±16
3	40.0	200	Break
4	40.0	200	Break

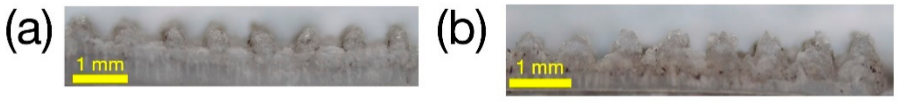

Figure S3. Side view of the Ecoflex micro-cones using the parameters of table S2

Table S3 CO_2 laser ablation parameters

Number of laser ablations	Laser power(W)	Laser Speed(mm/s)	Microstructure height(μm)
1	30	200	230±15
2	30	200	450±20
3	30	200	660±14
4	30	200	870±17

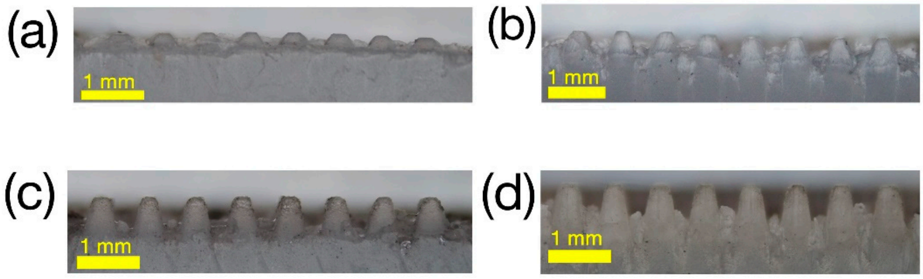

Figure S4. Side view of the ecoflex micro-cones using the parameters of table S3

Table S4 CO_2 laser ablation parameters

Laser power(W)	Laser Speed(mm/s)	Microstructure height (μm)
6	200	750±13
7	200	660±19
8	200	400±18

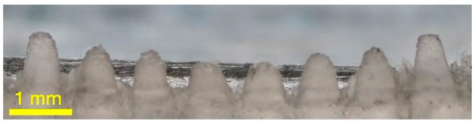

Figure S5. Side view of the Ecoflex micro-cones using the parameters of table S4

Table S5 CO_2 laser ablation parameters

Number of laser ablations	Laser power(W)	Laser Speed(mm/s)	Microstructure height(μm)
1	30	200	200±13
2	30	200	410±16
3	30	200	630±14
4	30	200	820±20

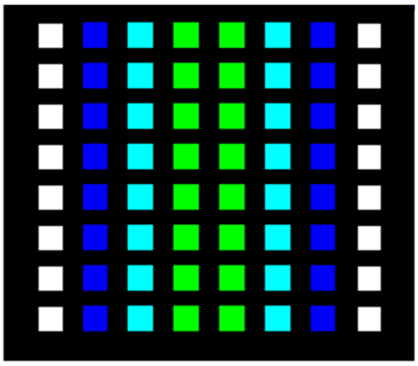

Figure S6. Scheme with gradient micro-cones architecture where 20 W of ablation power in the black area, 6 W in the dark blue area, 7 W in the light blue area and 8 W in the green area.

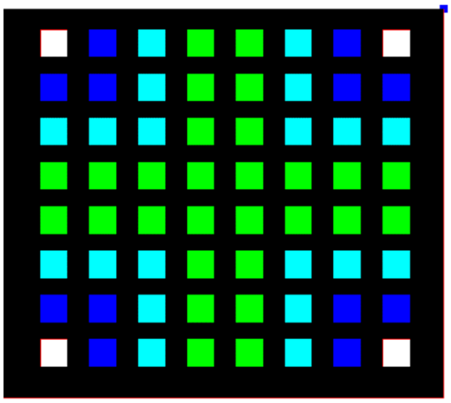

Figure S7. Scheme with gradient micro-cones architecture where 20 W of ablation power in the black area, 6 W in the dark blue area, 7 W in the light blue area and 8 W in the green area.

Table S6 CO_2 laser ablation parameters

Laser power(W)	Laser Speed(mm/s)	Microstructure height(μm)
6	200	750±14
7	200	630±16
8	200	390±15

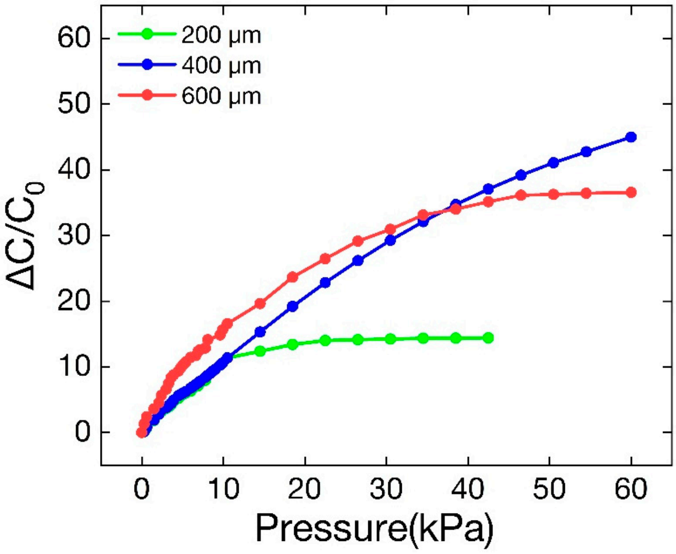

Figure S8. Relative capacitive response of sensors with different dielectric layer thicknesses

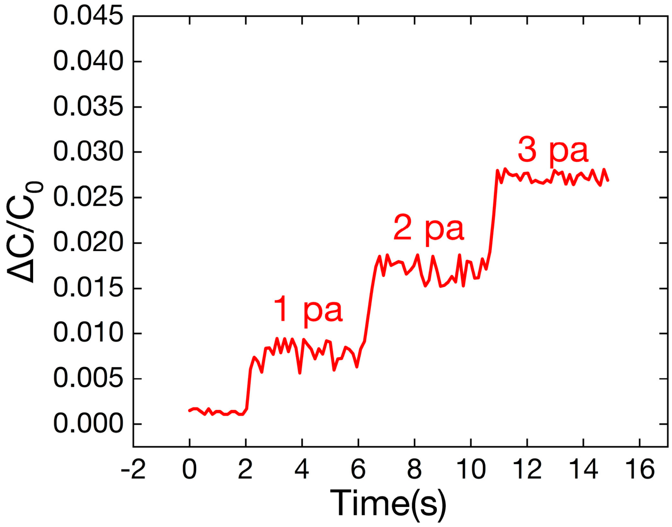

Figure S9. The limit of detection of sensor

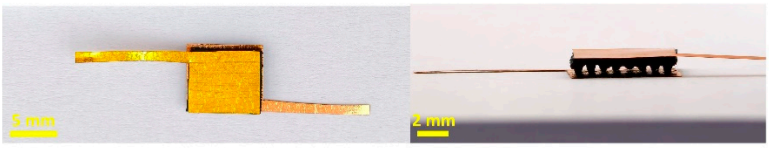

Figure S10. Top and side views of the finished sensor.

Table S7 Comparison of works in recent years

Micro structure	Fabrication Method	Mechanism	Linearity Range/kPa	Sensitivity /kPa^{-1}	Ref.
Tilted pillars	photoresist	Capacitive	0-2<br>2-14	0.42<br>0.04	[46]
Porous	Dissolved sugar	Capacitive	2-10	1.72	[32]
Porous	Solvent volatilizatio n	Capacitive	3-120	0.1	[47]
Porous	Anchored	Capacitive	0.5-4	0.04	[48]
Wrinkles	Heating shrinkage	Capacitive	0-10	0.148	[49]
Pyramids	photoresist	Capacitive	0-1<br>1-10	4.1<br>2.0	[50]
Cones	Lotus leaf mold	Capacitive	0-2<br>2-7<br>7-15	1.19<br>0.3<br>0.77	[51]
Nanosphere s	Spin nanosphere	Capacitive	0-2	1	[52]
Rough surface	Paper mold	Capacitive	0-2	0.62	[53]
Pyramidal	Si mold	Capacitive	0-20	0.022	[23]
Porous	Dissolved sugar	Capacitive	0-20	0.6	[54]
Porous	coated conductive layer	Capacitive	3-25	0.135	[55]
Wrinkles	Spontaneous formation	Capacitive	0-21	1.448	[37]
Cones	Laser ablation	Capacitive	0-60	0.75	This work
